# Peripheral Circulating Exosomal miRNAs Potentially Contribute to the Regulation of Molecular Signaling Networks in Aging

**DOI:** 10.3390/ijms21061908

**Published:** 2020-03-11

**Authors:** Hongxia Zhang, Kunlin Jin

**Affiliations:** Department of Pharmacology and Neuroscience, University of North Texas Health Science Center, Fort Worth, TX 76107, USA; Hongxia.Zhang@unthsc.edu

**Keywords:** exosomes, aging, serum, functional enrichment analysis, ingenuity pathway analysis, miRNA-mRNA networks, aging-related disease

## Abstract

People are living longer than ever. Consequently, they have a greater chance for developing a functional impairment or aging-related disease, such as a neurodegenerative disease, later in life. Thus, it is important to identify and understand mechanisms underlying aging as well as the potential for rejuvenation. Therefore, we used next-generation sequencing to identify differentially expressed microRNAs (miRNAs) in serum exosomes isolated from young (three-month-old) and old (22-month-old) rats and then used bioinformatics to explore candidate genes and aging-related pathways. We identified 2844 mRNAs and 68 miRNAs that were differentially expressed with age. TargetScan revealed that 19 of these miRNAs are predicated to target the 766 mRNAs. Pathways analysis revealed signaling components targeted by these miRNAs: mTOR, AMPK, eNOS, IGF, PTEN, p53, integrins, and growth hormone. In addition, the most frequently predicted target genes regulated by these miRNAs were EIF4EBP1, insulin receptor, PDK1, PTEN, paxillin, and IGF-1 receptor. These signaling pathways and target genes may play critical roles in regulating aging and lifespan, thereby validating our analysis. Understanding the causes of aging and the underlying mechanisms may lead to interventions that could reverse certain aging processes and slow development of aging-related diseases.

## 1. Introduction

Aging is a highly complex biological process that is often accompanied by a general decline in tissue function and an increased risk for aging-related diseases, such as cardiovascular disease, stroke, cancer, and neurodegenerative diseases. Indeed, as average lifespan continues to increase, aging-related functional decline, such as cognitive impairment, will likely become a health care priority [1]. For example, the most common form of dementia is Alzheimer’s disease (AD), but a large proportion of cognitive impairment cases in the aged population is not due to AD but rather to normal aging process. Thus, it is important to identify ways to maintain functional integrity during aging [2]. Many theories have been proposed to explain why we age [3]. Recently, we proposed a new theory positing that aging is the process of continuous impairment of microcirculation in the body [4]. Indeed, compelling evidence indicates that systemic factors in the blood profoundly reverse aging-related impairments [5,6,7], which are influenced by specific rejuvenating or deteriorating factors, e.g., proteins, microRNAs (miRNAs), and mRNAs [8]. Thus, many circulating factors have been identified as attractive biomarkers for tissue-specific diseases and aging [9,10]. However, the mechanisms underlying the contributions of blood-derived factors to aging remain unclear. 

Research over the last two decades has demonstrated that cells mainly communicate by releasing extracellular vesicles (EV) that can act on nearby cells (paracrine signaling) or end up in circulating body fluids, with possible effects at distant sites (endocrine signaling) [11]. Exosomes are small EVs (approximately 50–150 nm in diameter) of endosomal origin that initially form as intraluminal vesicles inside late endosomal compartments. Indeed, exosomes contain many specific proteins, mRNAs, miRNAs, and long noncoding RNAs [12] and play a vital role in cell communication by transferring their cargo between source and target cells, which is also important in aging and aging-related disease [13]. For example, injection of serum exosomes from young mice into old mice could alter the expression pattern of aging-associated molecules to mimic that of young mice [14]. In addition, studies have documented that exosomes from brain cells can cross the blood-brain barrier (BBB) and serve as peripheral circulating biomarkers of cognitive impairment in AD [15,16,17], and, blood exosomes can also cross the BBB to target brain cells and affect brain function [18,19,20,21]. Thus, peripheral circulating exosomes have diagnostic and therapeutic potential. However, most studies have focused on establishing exosomal protein or miRNA profiles for comparing disease states and matched controls, and few studies have focused on characterizing proteins and miRNAs in peripheral circulating exosomes during normal aging [22]. Therefore, it is critical to define the profiles for exosomal proteins and miRNAs that can be transferred from exosome to recipient cells. Importantly, it has been estimated that miRNAs regulate ~31% of all eukaryotic genes by promoting degradation of their mRNAs or inhibiting their translation [23,24]. Indeed, miRNA-mediated regulation governs metabolism, immunity, lifespan, cell proliferation, apoptosis, and development [25,26,27], as well as pathological processes such as cancer and cardiovascular and neurodegenerative disease [28,29,30]. Therefore, among the exosomal cargo that is transferred to recipient cells, miRNAs likely have the greatest downstream impact on cell functions. To explore the role of circulating exosomes in aging processes, exosomal miRNAs must be more broadly characterized. In addition, recent evidence suggests that numerous signaling pathways regulate normal aging processes. However, research is lacking concerning how aging affects co-expression profiles for exosomal miRNAs and mRNAs and how miRNA-mRNA regulatory networks systematically influence aging processes. 

To address shortcomings in our knowledge of exosomal miRNA functions, we used next-generation sequencing to establish miRNA and mRNA profiles for circulating exosomes isolated from young and old rats. We also investigated the possible role of exosomal miRNAs in aging by analyzing the biological importance of the miRNA targets and in major signaling pathways associated with aging using bioinformatic tools including Gene Ontology (GO) enrichment, Kyoto Encyclopedia of Genes and Genomes (KEGG) enrichment and pathways, eukaryotic orthologous groups (KOG) function classification, and Ingenuity Pathway Analysis (IPA). Our findings may provide a basis for understanding the physiological consequences of aging-related changes in the makeup of circulating miRNAs and could lead to potential interventions for aging-related diseases. 

## 2. Results

### 2.1. Characterization of Serum Exosomes

**Figure 1:**
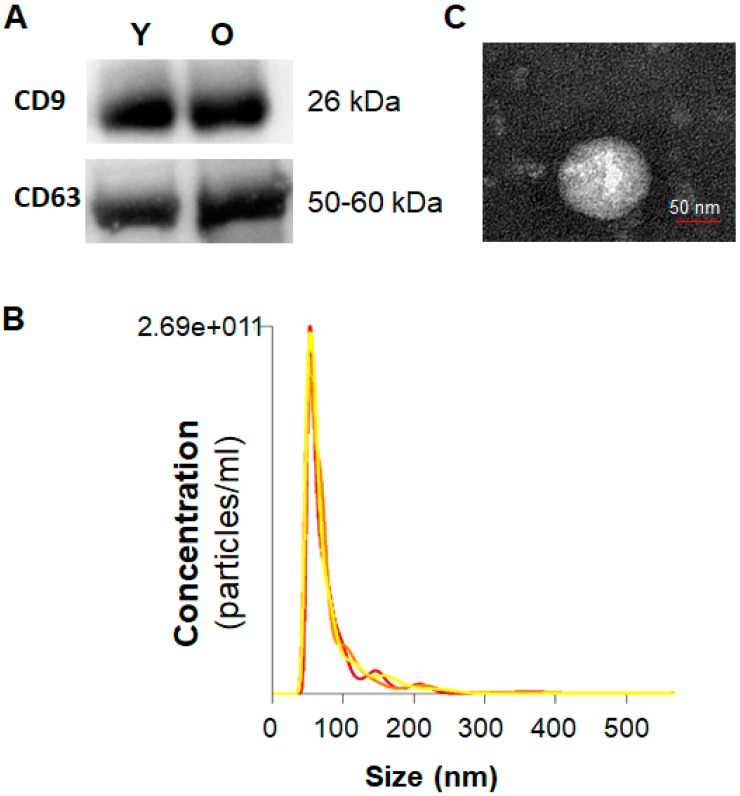
Characterization of serum exosomes. (**A**) Western blotting for CD63 and CD9 in serum exosomes isolated from young and old rats. (**B**) Average overall size distribution of exosomes from serum of old rats using the Nanoparticle Tracking Analysis. (**C**) Representative transmission electron microscopy image showing the typical morphology and size range of exosomes from serum of old rat. Y, serum exosomes from young rats; O, serum exosomes from old rats.

We first characterized the protein content of serum exosomes isolated from young and old rats using Western blotting. Serum exosomes from each of young and old rats were positive for the exosome markers, CD63 and CD9 (Figure 1A). Nanoparticle tracking analysis (NTA) (Figure 1B) verified a strong enrichment of particles in the range 40–120 nm, with mean size of 82 +/− 0.8 nm, supporting a multimodal size distribution of exosomes with a peak diameter of 70–120 nm, consistent with previous reports [31,32]. In addition, transmission electron microscopy (TEM) was used to confirm that the purified particles were membrane bound, round and heterogeneous in size (40–120 nm) (Figure 1C). 

### 2.2. Differentially Expressed RNAs in Serum Exosomes with Age

**Figure 2:**
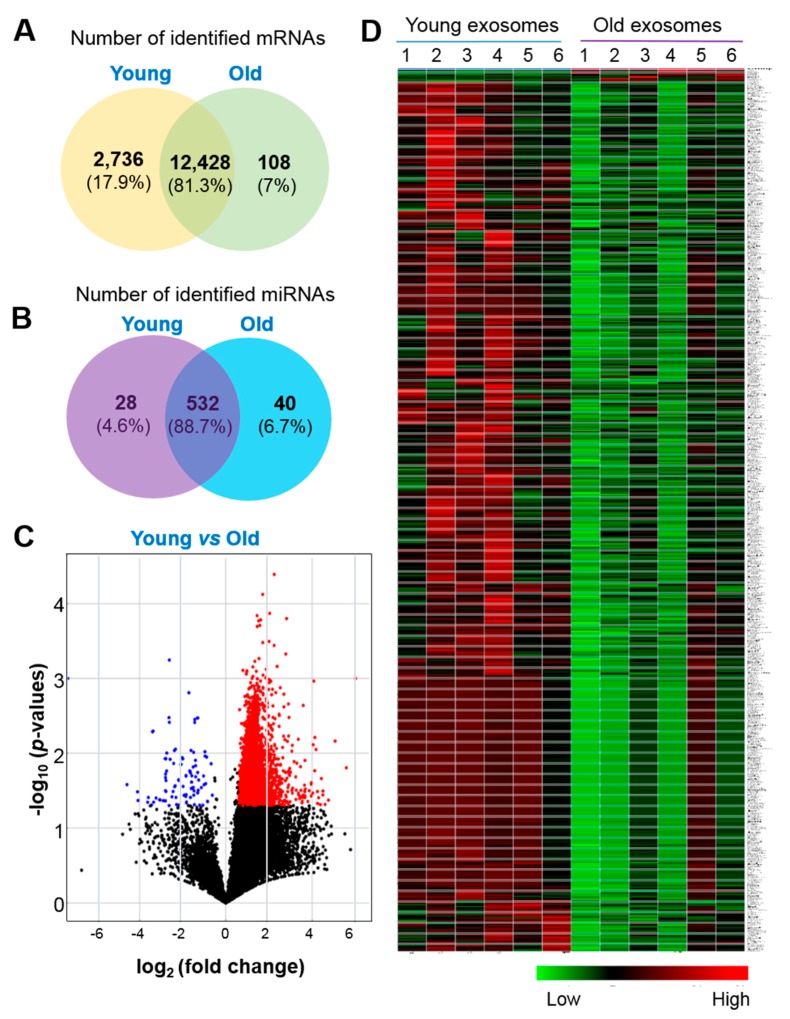
Profiles for mRNAs and miRNAs of serum exosomes from young and old rats. (**A**,**B**) Venn diagram of all differentially expressed mRNAs (**A**) and miRNAs (**B**) identified in serum exosomes. (**C**) Volcano plot for comparing the differentially expressed exosomal mRNAs and miRNAs in serum from young and old rats after analysis with TargetScan (fold change > 1.5 and *p* < 0.05). (**D**) Heatmap of the differentially expressed mRNAs and miRNAs in serum exosomes from young and old rats (*n* = 6 each group) after TargetScan (fold change > 1.5 and *p* < 0.05). Young, serum exosomes from young rats; Old, serum exosomes from old rats.

To determine whether aging affects the levels of serum exosomal RNAs, RNA profiles were determined by next-generation RNA Sequencing. After quality control and filtering, a total of 35117 RNAs, including mRNA, miRNAs and other type of RNAswere identified in exosomes from serum of young and old rats (Supplementary data). Following application of thresholds for significance, 2736 (17.9%) were down-regulated and 108 (7%) were up-regulated in serum exosomes from old rats (*p* < 0.05, >1.5-fold change; Figure 2A), among identified 15272 mRNA. In addition, 600 miRNAs were identified after quality control, among which 68 were relatively abundant in old rats, including 28 that were down-regulated and 40 that were up-regulated serum exosomes from old rats (*p* < 0.05, >1.5-fold change; Figure 2B). A volcano plot (Figure 2C) and cluster analysis (Figure 2D) revealed the overall distribution of differentially expressed mRNAs and miRNAs of serum exosomes with age after analysis with TargetScan.

### 2.3. Identification of miRNA-Targeted mRNAs 

**Figure 3:**
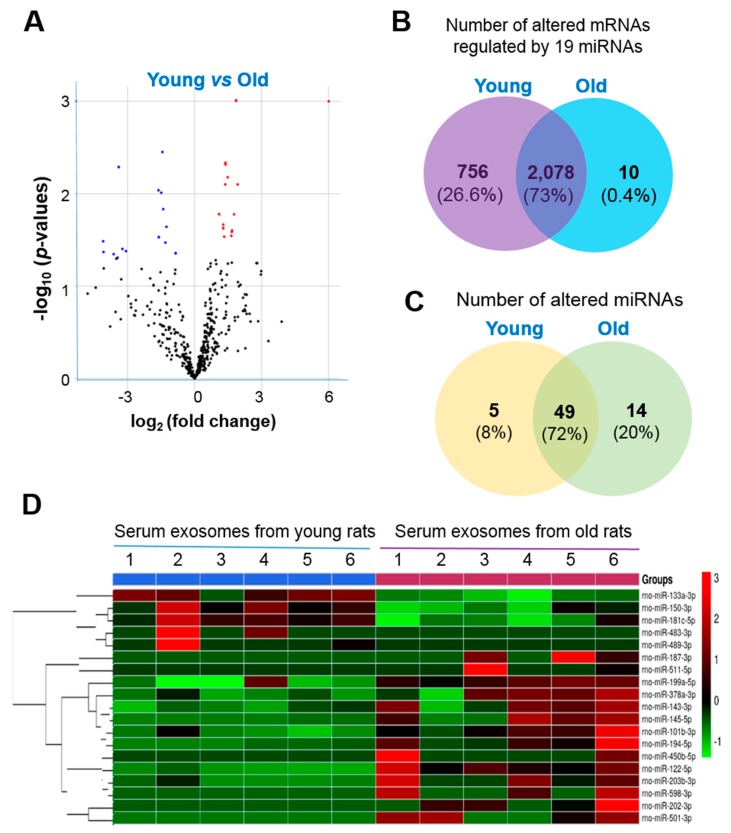
Profiles for differentially expressed miRNAs of serum exosomes from young and old rats. (**A**) Volcano plot showing the differentially expressed miRNAs (fold change > 1.5 and *p* < 0.05). (**B**) Venn diagram showing the differentially expressed mRNAs. (**C**) Venn diagram of the differentially expressed miRNAs. (**D**) Heat map of hierarchical clustering of 19 miRNAs that were identified in serum exosomes from young and old rats (*n* = 6 each group).

MiRNAs regulate expression of specific genes via hybridization to mRNAs to promote their degradation in order to inhibit their translation or both [33]. A volcano plot revealed the overall distribution of the exosomal miRNAs we identified in this study (Figure 3A). To study the possible functional roles of the differentially expressed miRNAs, their potential mRNA targets were analyzed with Targetscan. Among the 68 miRNAs, only 19 were associated with 766 of the 2844 mRNAs that were differentially expressed with age (Figure 3B), suggesting that these miRNAs contribute to the age-dependent regulation of specific mRNAs. Among them, 5 mRNAs were down-regulated and 14 were up-regulated in serum exosomes from old rats compared with those from young rats (Figure 3C). MiRNA-483-3p and miRNA-489-3p were detected only in exosomes from young rats, and miRNA-187-3p, miRNA-202-3p, miRNA-450b-5p, miRNA-501-3p, miRNA-511-5p, and miRNA-598-3p were detected only in exosomes from old rats (Table 1). Figure 3D presents results of a cluster analysis of differentially expressed miRNAs for each sample. 

### 2.4. GO Enrichment Analysis of miRNA-Targeted mRNAs

**Figure 4:**
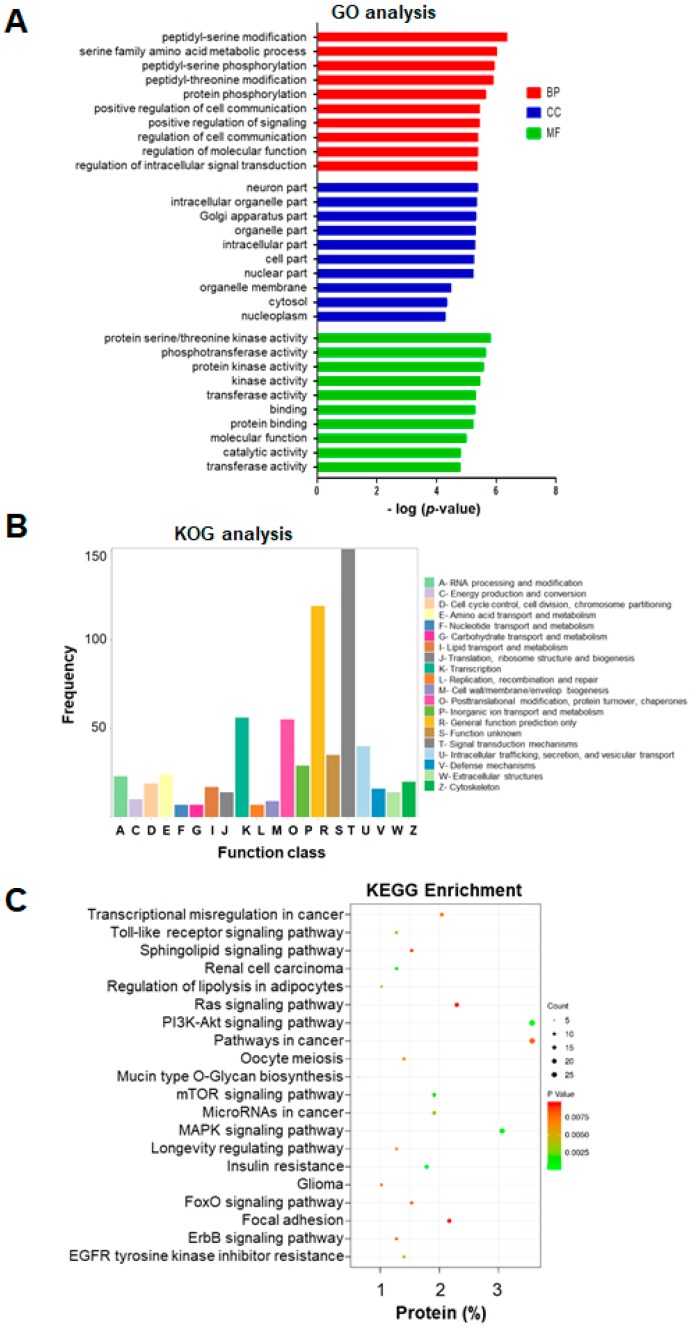
Gene Ontology (GO) analysis, eukaryotic orthologous groups (KOG) functional classification, and Kyoto Encyclopedia of Genes and Genomes (KEGG) pathway analysis of target genes (mRNAs) regulated by the 19 miRNAs that were differentially expressed between young and old rats. (**A**) GO annotation of predicted targets. The top 10 most enriched GO terms are listed in terms for biological process (BP), cellular component (CC), and molecular function (MF) based on *p*-values. (**B**) KOG functional classification of target genes. The vertical axis represents the frequency of target genes classified into the specific categories, and the horizontal axis represents the KOG functional classification. (**C**) The top 20 most common KEGG pathways of the differentially expressed mRNAs regulated by the 19 miRNAs. Fold change > 1.5 and *p* < 0.05. GO, gene ontology; KOG, eukaryotic orthologous groups; KEGG, Kyoto Encyclopedia of Genes and Genomes.

To gain a better understanding of the potential role of these exosomal miRNAs in aging, we used Blastp to carry out functional annotation and enrichment analysis of their target genes identified in the GO enrichment analysis. Functional annotation was categorized by biological process, cellular component and molecular function, and only the top 10 GO terms having the smallest *p*-value were considered. These categories represent the annotation of the functional enrichment of targeted genes, and a lower *p*-value represents a greater functional enrichment of a relative term. Notably, almost all the genes listed under these GO terms were downregulated in serum exosomes from old rats. This analysis revealed several enriched functional categories and target genes, including genes involved in the posttranslational modification of proteins, metabolic processes, cell communication, molecular function, and intracellular signal transduction (Figure 4A). 

### 2.5. KOG and KEGG Enrichment and Analyses

KOG was used to functionally classify mRNAs (766) regulated by the 19 exosomal miRNAs that were differentially expressed between young and old rats. Among the resultant 25 KOG classifications, genes involved in “signal transduction mechanisms” were the ones most commonly targeted (151 genes), followed by “general function prediction only” (119 genes), “transcription” (56 genes), “posttranslational modification and protein turnover” (55 genes), and “intracellular function and secretion and vesicular transport “(40 genes) (Figure 4B).

KEGG is a comprehensive knowledge base for both functional interpretation and practical application of genomic information [34]. KEGG pathway analysis identified 20 pathways that differed significantly (*p* < 0.05) between exosomes of young and old rats (Figure 4C). Among these pathways, the following were found to be involved in aging and lifespan: insulin resistance, mitogen-activated protein kinase (MAPK) signaling, PI3 kinase (PI3K)–Akt signaling, mammalian target of rapamycin (mTOR) signaling, toll-like receptor signaling, FoxO signaling, ErbB signaling, longevity-regulating signaling, and resistance to inhibitors of epidermal growth-factor receptor (EGFR) tyrosine kinase (Figure 4C). 

### 2.6. Analysis of Pathways and Interaction Networks 

**Figure 5:**
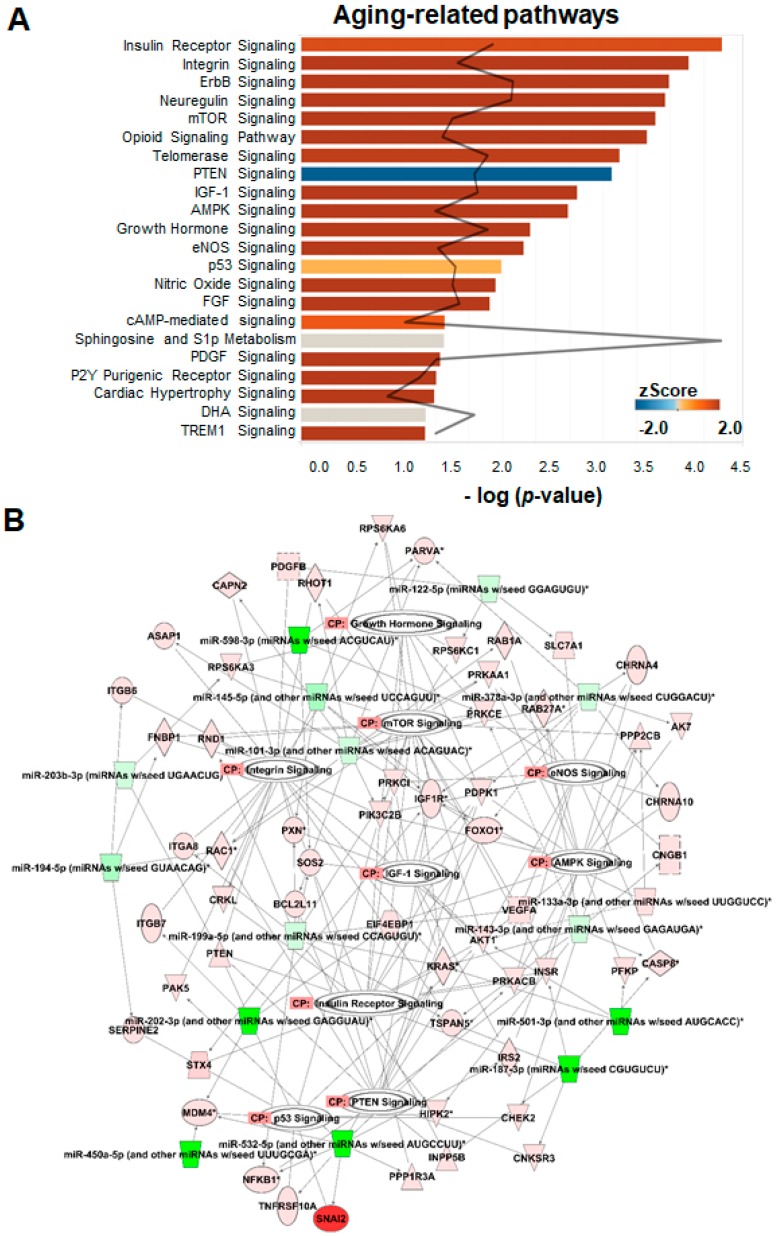
Ingenuity Pathway Analysis (IPA) of the differentially expressed miRNAs in serum exosomes from young and old rats. (**A**) IPA showing the 22 most significant aging-related pathways involving mRNAs, whose expression is regulated by differentially expressed miRNAs in serum exosomes from young and old rats. Each Z score represents the upregulation or downregulation of gene expression based on young vs. old. The black curve denotes the ratio between the number of the differentially expressed target genes and the total number of genes in each of these pathways. (**B**) IPA-predicted network for the differentially expressed miRNAs showing predicted targets and their association with biological functions in aging-related signaling pathways governed by the following factors: growth hormone signaling, mammalian target of rapamycin (mTOR) signaling, endothelial nitric oxide synthase (eNOS) signaling, integrin signaling, insulin-like growth factor-1 (IGF-1) signaling, AMP-activated protein kinase (AMPK) signaling, insulin receptor signaling, p53 signaling, and phosphatase and tensin homolog 10 (PTEN) signaling.

We then carried out IPA for molecular pathways associated with serum exosomal miRNAs during aging. The results showed that 163 IPA canonical pathways were predicted to be significantly related to the expression of serum exosomal miRNAs, based on *p* < 0.05. The top 22 most strongly aging-associated pathways targeted by miRNAs are shown in Figure 5A. Those discovered aging-related signaling pathways included insulin, integrin, ErbB, neuregulin, mTOR, opioid, telomerase, phosphatase and tensin homolog 10 (PTEN), insulin-like growth factor-1 (IGF-1), adenosine monophosphate-activated protein kinase (AMPK), growth hormone, endothelial nitric oxide synthase (eNOS), nitric oxide, fibroblast growth factor (FGF), cyclic adenosine monophosphate (cAMP), sphingosine, platelet-derived growth factor (PDGF), docosahexaenoic acid (DHA), triggering receptor expressed on myeloid cells 1 (TREM1), and p53, suggesting that miRNAs target multiple biological pathways that modulate aging. 

**Figure 6:**
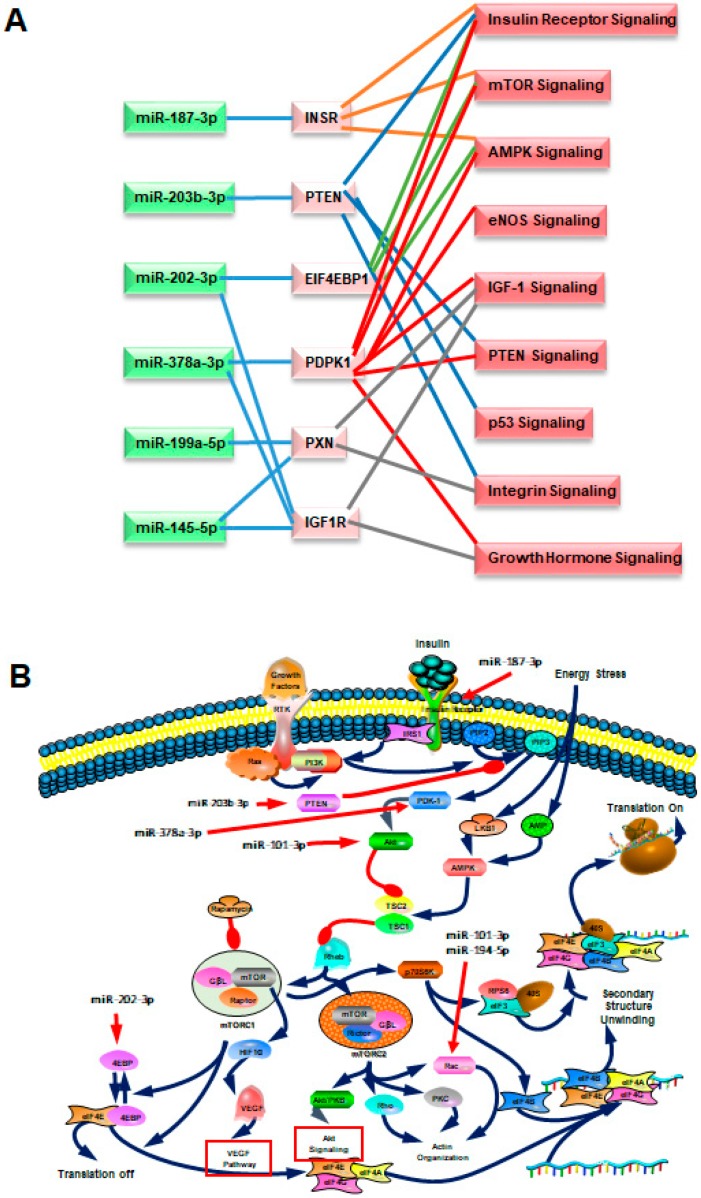
The most common target genes and mTOR pathway regulated by the differentially expressed exosomal miRNAs. (**A**) *EIF4EBP1, INSR, PDPK1, PTEN, PXN*, and *IGF-1R* are the most common network genes targeted by the prominent circulating exosomal miRNAs including miR-187-3p, miR-203b-3p, miR-202-3p, miR-378a-3p, miR-199a-5p, and miR-145-5p. (**B**) IPA networks showing the regulatory effects of the differentially expressed miRNAs from rat serum on mTOR signaling.

Figure 5B presents the IPA network results, and Table 2 lists the miRNAs involved in the nine pathways. Similar to IPA results, the networks contained genes predicted to be involved in metabolism, growth hormone signaling, and oxidative stress. As shown in Figure 5B, each pathway was linked with several gene transcripts, and individual genes could be regulated by several miRNAs. This suggested that the serum exosomal miRNAs that regulate crosstalk between pathways differ among young and old rats. The most common proteins in the networks were eukaryotic translation initiation factor 4E binding protein 1 (EIF4EBP1), insulin receptor (INSR), phosphoinositide dependent protein kinase 1 (PDPK1), PTEN, paxillin (PXN), and IGF-1 receptor (IGF-1R) that were targeted by the most prominent miRNAs (Figure 6A). Overall, the results establish putative functions between miRNAs and their target mRNAs, molecular networks, and biological pathways that modulate the makeup of serum exosomal miRNAs in young versus old animals. Figure 6B shows one such example of miRNA-mediated regulation. 

## 3. Discussion

The past two decades have witnessed the use of heterochronic blood exchange techniques, including heterochronic parabiosis, heterochronic blood or plasma transfer, or heterochronic apheresis, as tools for studying the biology of aging. Indeed, heterochronic blood exchange from a young to an old animal resulted in rejuvenation, whereas accelerated aging in a young animal was observed after heterochronic blood exchange from an old animal [5]. To explore the underlying mechanism, we used Exo-NGS analysis to compare the expression profiles for mRNAs and miRNAs in serum exosomes isolated from young and old rats. We Identified 68 miRNAs and 2844 mRNAs in serum exosomes that were differentially expressed between young and old rats. In contrast to mRNAs, little is known about changes in miRNA abundance in the aging process. For this reason, we focused on circulating miRNAs, which serve as potential biomarkers and therapeutic targets for aging-related disease. To determine how these circulating miRNAs affect aging, it is important to identify the targets for each miRNA. Our data revealed that, of the 68 differentially expressed serum exosome miRNAs we identified, 19 were predicated to target 766 differentially expressed mRNAs based on TargetScan analysis. Among the 19 miRNAs, 14 were more abundant in exosomes from old rats than from young rats, and five were less abundant. These results are consistent with reports that the abundance of the majority of these 14 miRNAs including miR-150-3p, miR-378-3p, miR-199a-5p, miR-145-5p, miR-598-3p, miR-122-5p, miR-194-5p, miR-203a-3p, miR-202-3p, miR-145-5p, and miR-532-5p, was elevated in blood or tissue samples from older humans, mice and rats [17,35,36,37,38,39,40,41]. Our data also confirmed that miR-181a-5p and miR-133a-3p decreased with age [40,42]. These 14 miRNAs have been linked with aging, and the expression of some of them has been associated with cancer, longevity, inflammatory responses, and aging-related neurodegenerative and cardiac diseases [17,36,37,38,39,40,41,42,43,44,45,46]. Collectively, the abundance of the majority of our differentially expressed miRNAs has been previously reported to be altered with age, suggesting roles for these miRNAs in lifespan. Interestingly, downregulation of miR-181a-5p in serum exosomes from old rats correlates negatively with the expression of pro-inflammatory cytokines IL-6 and TNFα and correlates positively with that of the anti-inflammatory cytokines TGFβ and IL-10 in the serum of rhesus monkeys [42]. Notably, the abundance of IL-6 and TNF-α has been correlated with aging [47]. Therefore, certain exosomal miRNAs may contribute to aging by regulating systemic inflammation, and the makeup of these miRNAs may serve as a biological signature of aging. 

We used Blastp and GO to functionally annotate miRNA-regulated genes and, identified biological processes that are altered by changes in exosomal miRNAs abundance changes with age. Among these processes, the most highly represented and enriched terms were protein posttranslational modification, metabolic process, cell communication, molecular function, and intracellular signal transduction, implying that these miRNAs may provide a significant link between aging and multiple biological processes through their regulation of target genes [48]. KEGG pathway analysis revealed that the mRNAs targeted by these miRNA targets were enriched in known aging-related signaling pathways [49,50,51]. The GO and KEGG analysis also revealed that most of the miRNA-targeted mRNAs are involved in signaling pathways and biological processes, that are critical for aging, suggesting that circulating miRNAs may help regulate the rate of aging and therefore are potential biomarkers for aging. Any individual miRNA may have the potential to act on numerous target genes, and therefore, multiple miRNAs have the potential to modulate numerous biological pathways. Hence, the impact of miRNAs on any particular pathway(s) can be assessed most effectively by examining any synergism between the miRNAs [52]. To further investigate how any single miRNA-mRNA interaction regulates aging-related pathways, we performed IPA and found that the altered circulating miRNAs target the signaling pathways governed by insulin, integrin, mTOR, AMPK, PTEN, IGF-1, growth hormone, eNOS and p53, which are crucial pathways in aging and lifespan [49,50,51]. For example, we found that miRNA-187-3p can regulate INSR mRNA and that miRNA-378a-3p andmiRNA-202-3p can regulate IGF-1R mRNA. Studies have documented an inverse correlation between cellular miRNA-187 levelss and glucose-stimulated insulin secretion [53] and that miRNA-378a may play a role in insulin resistant and the consequent of obesity [54]. It is well documented that the insulin/IGF-1 pathway plays a critical role in aging and longevity across a wide spectrum of species [55,56,57]. Evidence includes that either reducing the level of circulating IGF-1 or reducing the expression of IGF-1R increases longevity [57]; moreover, the loss of one allele of the *Igf-1* receptor increases the lifespan of mice by 33% [58]. We also found that miR-187-3p, miR-202-3p, and miR-378a-3p regulate the mRNA levels of *INSR, EIF4EBP1* and *PDK1*, the genes for which are targeted by) mTOR signaling pathway. The mTOR pathway integrates both intracellular and extracellular signals and serves as a central regulator of cell metabolism, proliferation and survival, and it also controls lifespan by regulating translation through activation of p70S6K and inhibition of the translation repressor eIF4EBP [59]. For example, knocking down three translational regulators, namely eIF4E, eIF4G, and eIF2B homologs, in *C. elegans* extends worm lifespan [60,61,62], and modulation of the translation of their mRNAs by a dominant-negative form of TOR extends lifespan [63]. Recent studies have shown that the lifespan of different mouse strains can be extended significantly when mTOR inhibitor of rapamycin is administrated [64,65]. There is no clear explanation how a reduction in signaling via mTOR or insulin/IGF-1 affects lifespan. However, one potential explanation is that global mRNA translation is reduced after inhibiting either of these signaling pathways, which may reduce the burden and energetic demands associated with protein folding, repair, and degradation, thus maintaining better overall protein homeostasis [51]. Our findings support this hypothesis. 

In addition to the insulin/IGF-1 and mTOR pathways, many other signaling pathways, such as the PTEN pathway, also modulate lifespan [66]. Indeed, PTEN has significant implications for extending human longevity through its antioxidant activity and contribution to the benefits of caloric restriction as well as its involvement DNA-damage reduction, inhibition of DNA replication, and tumor suppression [67]. We found that miR-203b-3p can target PTEN. Notably, signaling pathways, such as the insulin/IGF-1, mTOR and PTEN pathways, may individually regulate aging and lifespan. However, these signaling networks are not autonomous but connected through some specific mediators. For instance, mTOR has two complexes, namely mTOR complex 1 (mTORC1) and mTOR complex 2 (mTORC2) [59]. mTORC1 is regulated by Akt, and mTORC2 is an Akt activator [68]. PI3 kinase signaling activates mTORC2, which in turn activates a number of other kinases, including PKCα. Consistently, we found that the EIF4EBP1, INSR, PDPK1, PTEN, PXN, and IGF1R overlap and are regulated by at least two circulating miRNAs, and each of these pathways may play a unique role in aging [49,50,51]. 

Taken together, our findings suggest that changes in the makeup of circulating exosomal miRNAs with age not only can be considered as a potential predictor of animal age but also may contribute to aging via several key signaling pathways that regulate aging and lifespan. It will be important to identify and understand the mechanisms of rejuvenation and accelerated aging, because the findings concerning rejuvenation can potentially reverse deleterious processes of aging, whereas the findings concerning accelerated aging may pinpoint potential pathways for interventions that may slow the rate of aging and the incidence of aging-related disease. The challenge for the future will be to determine how these mediators map onto the different pathways and interact with each other, and to decipher how they contribute to the molecular mechanisms in aging. 

## 4. Materials and Methods

### 4.1. Isolation of Serum Exosomes

Whole blood was collected from young (three-month-old) or old (22-month-old) rats (*n* = 6 per group) via cardiac puncture into BD Vacutainer® Plus Glass Serum blood collection tubes (Becton Dickinson, NJ, USA). Whole blood samples were allowed to clot by standing at room temperature for 30 min, and the clots were removed by centrifugation for 10 min at 1000× *g* at 4 °C. The isolated serum samples were aliquoted and stored at −80 °C. 

Serum exosomes from young or old rats were isolated using the ExoQuick Exosome precipitation kit (System Biosciences, CA, USA). Briefly, serum (500 µL) was centrifuged at 3000× *g* for 15 min at 4 °C to eliminate cells and cell debris. The supernatant was transferred to a sterile micro-tube, and an appropriate volume of exosome precipitation solution from the kit was added, with incubation for 30 min at 4 °C. The mixture was then centrifuged at 1500× *g* for 30 min at 4 °C, and the exosome pellet was re-suspended in sterile phosphate-buffered saline at 4 °C. 

### 4.2. Characterization of Serum Exosomes

Both the concentration and average size of the isolated serum exosomes were determined by nanoparticle-tracking analysis (NTA) using the Exosome Nanosight Analysis Service of System Biosciences (Palo Alto, CA, USA). The serum exosomes were also observed using transmission electron microscopy (TEM, FEI Tecnai G2 Spirit BioTwin, OR, USA) to determine morphology and the extent of dispersion, this analysis was performed at the Electron Microscopy Core Facility at the University of Texas Southwestern Medical Center, TX, USA. The enrichment of exosomes was determined by Western blotting using antibodies against exosomes components such as CD63 andCD9.

### 4.3. Western Blotting

Serum exosomes were lysed in RIPA buffer and the protein concentration was determined using the Quick Start Bradford protein assay (Pierce™ BCA Protein Assay kit, Thermo Fisher Scientific, MA, USA). The lysates (10 µg) were electrophoresed through 8–12% SDS-PAGE gels, and the separated proteins were transferred to a nitrocellulose membrane. The membrane was incubated in blocking buffer (5% milk in Tris-buffered saline with 0.05% *w*/*v* Tween-20) for 1 h at room temperature and then incubated overnight at 4 °C with mouse antibody against rat CD63 (1:1000, BD Pharmingen, CA, USA) and CD9 (1:1000, BD Pharmingen). Immunopositivity was detected with a horseradish peroxidase (HRP)—conjugated secondary antibodies and the Pierce enhanced chemiluminescence (ECL) substrate (Thermo Fisher Scientific, MA, USA). The data were recorded and analyzed using the ChemiDoc Imaging System (Bio-Rad). 

### 4.4. Isolation of Total RNA fromEexosomes and Next-Generation RNA Sequencing

Total RNA was isolated from serum exosomes using the SeraMir Exosome RNA Purification Column kit (System Biosciences, CA, USA). For each sample, 1 µL of the final RNA eluate was used for measurement of RNA concentration with the Agilent Bioanalyzer Small RNA Assay using the Bioanalyzer 2100 Expert instrument (Agilent Technologies, Santa Clara, CA, USA). Serum exosomal RNAs (N = 6 each group) were sent to the Exo-NGS^™^ (Exosomal RNA-Seq) services for next-generation RNA sequencing (System Biosciences, CA, USA) using small RNA libraries. Next-generation RNA sequencing was performed on an Illumina NextSeq instrument (Illumina, CA, USA) with 1 × 75 bp single-end reads at an approximate depth of 10–15 million reads per sample.

### 4.5. Data Processing

Raw data were analyzed using an integrated UCSC genome browser on the Banana Slug analytics platform (UCSC, CA, USA). Briefly, the exosome Small RNA-seq Analysis kit was initiated with a data quality check of each input sequence using FasQC (Wellcome Sanger Institute, UK) an open-source quality control tool for analyzing high-throughput sequence data. Following the quality-control step, the RNA-seq reads were processed to detect and remove unknown nucleotides at the ends of reads, trim sequencing adaptors, and filter reads for quality and length, using FastqMcf, which is part of the EA-utils package (ExpressionAnalysis, NC, USA) and PRINSEQ (http://prinseq.sourceforge.net/, USA). FastQC was then repeated to analyze the trimmed reads, thus allowing a before and after comparison. Sequence reads in the improved set were mapped to the reference genome using Bowtie, an ultrafast, memory-efficient short-read aligner. Expression analyses, including computation of read coverage and noncoding RNA abundance, were performed using the open-source software SAMtoolsand Picard (Github, CA, USA).

### 4.6. Bioinformatics Analysis 

After data processing, expression statistics for the normalized reads were evaluated using analysis of variance to identify differentially expressed genes. Differentially expressed genes were selected if the fold changes (FC) in expression was > 1.5 with a *p*-value < 0.5. From the data set of miRNAs and mRNAs for which expression was significantly altered between serum exosomes isolated from young and old rats, the potential regulation of a mRNA by a particular miRNA was predicted with TargetScan (http://www.targetscan.org/, MIT, MA, USA). The paired miRNAs and mRNAs were used for further analysis. 

Hierarchical cluster analysis (HCA) is an algorithmic approach to identify groups with varying degrees of (dis)similarity in a data set represented by a (dis)similarity matrix. This analysis was carried out with the Pheatmap package (https://CRAN.R-project.org/package=pheatmap, Estonia). The volcano plot is a type of scatter-plot that can quickly identify changes in individual data in large data-sets composed of replicate data; ggplot2 package (http://ggplot2.org, New Zealand) was used for this purpose. 

### 4.7. Gene Ontology (GO) and Pathway Enrichment Analysis

The biological function of each protein was annotated with Blastp (Blast2GO version 5, BioBam Bioinformatics, Spain) using the entire gene-expression database, and subsequent mapping was carried out with the GO database (www.geneontology.org/, Gene ontology resource, USA). To further understand the biological significance of differentially expressed exosome proteins, pathway analysis was carried out with the KEGG Orthology-Based Annotation System (http://kobas.cbi.pku.edu.cn, China). The association of proteins with different pathways was computed using the KEGG database (www.genome.jp/kegg, Japan). EuKaryotic Orthologous Groups (KOG) Analysis was based on the phylogenetic classification of proteins encoded in the complete genomes (www.ncbi.nlm.nih.gov/COG/, NCBI, MD, USA) project. IPA (QIAGEN, Germany) was used for additional functional annotation, including top canonical pathway, top disease and function and molecular and cellular functions, prediction of upstream, regulator effectors, and miRNA–mRNA relationship and interaction network analysis. 

### 4.8. Statistical Analysis

For the GO analysis, statistically significant alterations in functions of differentially expressed exosome proteins were assessed with Fisher’s exact test in Blast2GO with an adjusted *p*-value (false discovery rate, FDR) of <0.05 and fold change > 1.5. The statistical significance of changes in pathways identified with IPA was assessed with the right-tailed Fisher’s Exact test. A *p*-value of < 0.05 implies that the relationship of a set of targeted molecules and a process/pathway/transcription was randomly matched. A *Z* score of ≥ 2 or ≤ −2 indicated significant activation or significant inhibition, respectively. For all analysis, the difference was considered significant for *p* < 0.05. 

## Figures and Tables

**Table 1:** List of circulating miRNAs for which expression differed with age.

ID	*p*-Value	Fold Change	Expression Level (Old vs. Young)
rno-miR-101b-3p	0.0295919	3.0300921	Up
rno-miR-122-5p	0.005130112	10.50495019	Up
rno-miR-133a-3p	0.000973173	−3.631787956	Down
rno-miR-143-3p	0.033831212	2.469145377	Up
rno-miR-145-5p	0.032740298	17.04524832	Up
rno-miR-150-3p	0.016694482	−3.404719964	Down
rno-miR-181c-5p	0.006634803	−2.79454293	Down
rno-miR-187-3p	0.001	64	Up
rno-miR-194-5p	0.042745612	16.8112814	Up
rno-miR-199a-5p	0.009166599	3.06058214	Up
rno-miR-202-3p	0.001	64	Up
rno-miR-203b-3p	0.03954997	9.395112765	Up
rno-miR-378a-3p	0.043871243	1.797380304	Up
rno-miR-450b-5p	0.001	64	Up
rno-miR-483-3p	0.001	−64	Down
rno-miR-489-3p	0.001	−64	Down
rno-miR-501-3p	0.001	64	Up
rno-miR-511-5p	0.001	64	Up
rno-miR-598-3p	0.001	64	Up

**Table 2:** IPA of genes targeted by 19 miRNAs that were differentially expressed with age.

Ingenuity Canonical Pathways	−log (*p*-Value)	Related miRNA	Target Genes	Full Name
Insulin receptor signaling	4.48	miR-378a-3p	*PDK1*	3-phosphoinositide-dependent protein kinase 1
		miR-187-3p	*INSR*	insulin receptor
		miR-202-3p	*4E-BP1*	Eukaryotic translation initiation factor 4E-binding protein 1
		miR-101b-3p	*AKT*	RAC-alpha serine/threonine-protein kinase
		miR-199a-5p	*STX4*	Syntaxin-4
		miR-203b-3p	*PTEN*	phosphatase and tensin homolog deleted on chromosome
mTOR signaling	3.77	miR-378a-3p	*PDK1*	3-phosphoinositide-dependent protein kinase 1
		miR-187-3p	*INSR*	insulin receptor
		miR-202-3p	*eIF4E-BP1*	Eukaryotic translation initiation factor 4E-binding protein 1
		miR-101b-3p	*AKT*	RAC-alpha serine/threonine-protein kinase
		miR-194-5p	*RAC*	Aryl-hydrocarbon-interacting protein-like 1
AMPK signaling	2.84	miR-378a-3p	*PDK1*	phosphoinositide-dependent kinase-1
		miR-187-3p	*INSR*	insulin receptor
		miR-202-3p	*eIF4E-BP1*	Eukaryotic translation initiation factor 4E-binding protein 1
		miR-101b-3p	*AKT*	RAC-alpha serine/threonine-protein kinase
eNOS signaling	2.37	miR-378a-3p	*PDK1*	phosphoinositide-dependent kinase-1
		miR-187-3p	*CASP8*	caspase-8
		miR-101b-3p	*AKT*	RAC-alpha serine/threonine-protein kinase
		miR-122-5p	*CAT1*	cationic amino acid transporter 1
		miR-143-3p	*CASP8*	caspase-8
IGF-1 signaling	2.94	miR-378a-3p	*PDK1, IGF-1R*	phosphoinositide-dependent kinase-1, Insulin-like growth factor 1 receptor
		miR-202-3p	*IGF-1R*	Insulin-like growth factor 1 receptor
		miR-101b-3p	*AKT*	RAC-alpha serine/threonine-protein kinase
		miR-145-5p	*IGF-1R, PXN*	insulin-like growth factor 1 receptor, Paxillin
		miR-199a-5p	*PXN*	paxillin
PTEN signaling	3.31	miR-378a-3p	*PDK1*	phosphoinositide-dependent kinase-1
		miR-101b-3p	*BCL2L11, AKT*	bcl-2-like protein 11, RAC-alpha serine/threonine-protein kinase
		miR-203b-3p	*PTEN*	phosphatase and tensin homolog deleted on chromosome
		miR-532-5p	*NF-* *ƙB*	Nuclear factor NF-kappa-B
p53 signaling	2.13	miR-202-3p	*MDM4*	protein Mdm4
		miR-101b-3p	*AKT*	RAC-alpha serine/threonine-protein kinase
		miR-203b-3p	*PTEN*	phosphatase and tensin homolog deleted on chromosome
		miR-532-5p	*MDM4, Slug*	Protein Mdm4, Zinc finger protein SNAI2
		miR-450b-5p	*MDM4*	Protein Mdm4
		miR-194-5p	*PAI-1*	Glia-derived nexin
		miR-199a-5p	*HIPK2*	Homeodomain-interacting protein kinase 2
		miR-143-3p	*HIPK2, Chk2*	Homeodomain-interacting protein kinase 2, Serine/threonine-protein kinase Chk2
Integrin signaling	4.13	miR-378a-3p	*PARVIN-α*	parvin alpha
		miR-101b-3p	*ASAP1, PARVIN-α, AKT*	arf-GAP with SH3 domain, ANK repeat and PH domain-containing protein 1, parvin alpha, RAC-alpha serine/threonine-protein kinase
		miR-145-5p	*PXN, CRKL*	Paxillin, Crk-like protein
		miR-122-5p	*PDGFβ*	Platelet-derived growth factor subunit B
		miR-203b-3p	*PTEN*	phosphatase and tensin homolog deleted on chromosome
		miR-598-3p	*PARVIN-α*	parvin alpha
		miR-199a-5p	*PXN*	Paxillin
Growth hormone signaling	2.44	miR-378a-3p	*PDK1*	phosphoinositide-dependent kinase-1
		miR-202-3p	*IGF-1R*	Insulin-like growth factor 1 receptor
		miR-145-5p	*IGF-1R*	Insulin-like growth factor 1 receptor

## Supplementary Materials

Supplementary materials can be found at https://www.mdpi.com/1422-0067/21/6/1908/s1.
